# Genetic susceptibility to idiopathic membranous nephropathy in high-prevalence Area, Taiwan

**DOI:** 10.7603/s40681-014-0009-y

**Published:** 2014-08-06

**Authors:** Shih-Yin Chen, Cheng-Hsu Chen, Yu-Chuen Huang, Chia-Jung Chan, Da-Chung Chen, Fuu-Jen Tsai

**Affiliations:** 1 Genetics Center, Department of Medical Research, China Medical University Hospital, Taichung, Taiwan; 2 Department of Pediatrics, China Medical University Hospital, Taichung, Taiwan; 3 Department of Medical Genetics, China Medical University Hospital, No. 2, Yuh Der Road, 404, Taichung, Taiwan; 4 Graduate Institute of Chinese Medical Science, China Medical University, Taichung, Taiwan; 5 Division of Nephrology, Department of Internal Medicine, Taichung Veterans General Hospital, Taichung, Taiwan; 6 Department of Biotechnology and Bioinformatics, Asia University, Taichung, Taiwan; 7 Taiwan LandSeed Hospital, Pingjen City, Taoyuan, Taiwan; 8 Department of Chemical and Materials Engineering, National Central University, Taoyuan, Taiwan

**Keywords:** Membranous glomerulonephritis (MN), Single nucleotide polymorphisms (SNPs), Haplotype

## Abstract

Idiopathic membranous nephropathy (MN) is one common cause of idiopathic nephrotic syndrome in adults; 25% of MN patients proceed to end-stage renal disease. In adults, membranous nephropathy is a lead cause of nephrotic syndrome, with about 75% of the cases idiopathic. Secondary causes include autoimmune disease, infection, drugs and malignancy. Three hypotheses about pathogenesis have surfaced: preformed immune complex, *in situ* immune complex formation, and auto-antibody against podocyte membrane antigen. Pathogenesis does involve immune complex formation with later deposition in sub-epithelial sites, but definite mechanism is still unknown. Several genes were recently proven associated with primary membranous nephropathy in Taiwan: *IL-6, NPHS1, TLR-4, TLR-9, STAT4,* and *MYH9* . These may provide a useful tool for diagnosis and prognosis. This article reviews epidemiology and lends new information on *KIRREL2* (rs443186 and rs447707) polymorphisms as underlying causes of MN; polymorphisms revealed by this study warrant further investigation.

## 1. Introduction

Idiopathic membranous nephropathy (MN), common cause of nephrotic syndrome, accounts for about 40% of adult cases with clinical presentation of severe proteinuria, edema, hypoalbuminuria and hyperlipidemia [1]. Its characteristics include basement membrane thickening and subepithelial immune deposits without cellular proliferation or infiltration [2]. Prior study suggested MN as causing chronic kidney disease (CKD) and as final result of end-stage renal disease (ESRD) [3]. Therapy such as nonspecific antiproteinuric measures and immunosuppressive drugs yielded disappointing results, heightening interest in new therapeutic targets [4]. Taiwan has the highest prevalence of ESRD worldwide; MN may be one cause [5-7]. Genetic and environmental factors may contribute to progression and renal fibrosis in most renal diseases. Identifying genetic mechanisms related to high incidence of MN is crucial to current situation in Taiwan. This review highlights candidate genes studied over these past three years in Taiwan and discusses their implications in MN pathogenesis.

## 2. Genetic association studies on MN over three years in Taiwan

**Fig. 1:**
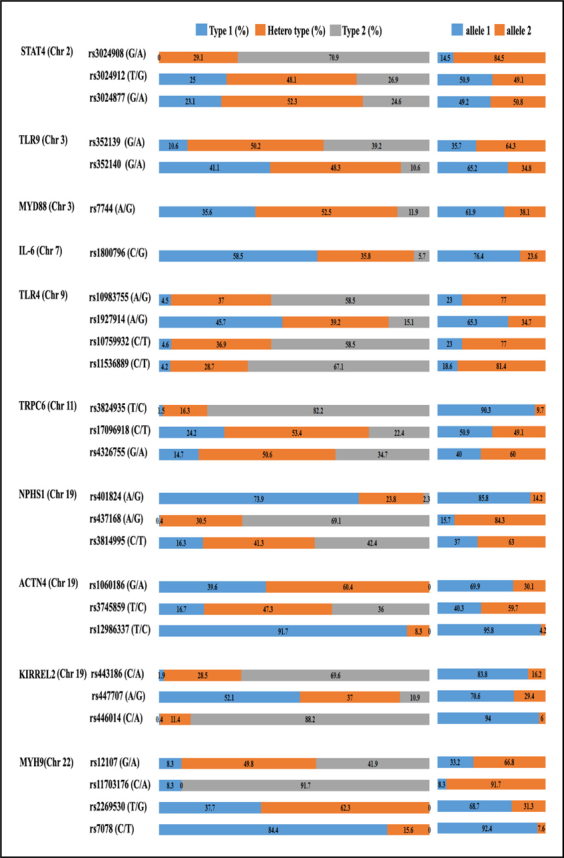
Distributions of genotypic and allelic frequencies of polymorphisms in normal population in Taiwan.

**Fig. 2:**
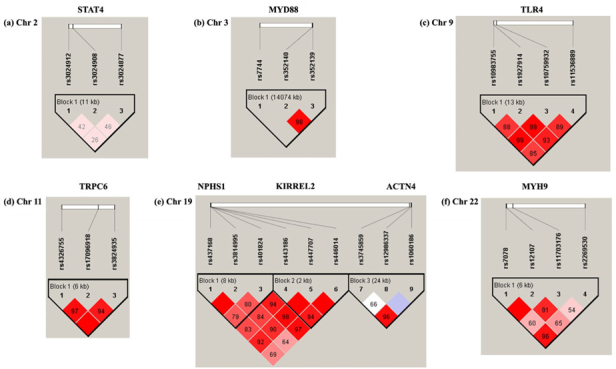
LD and haplotype block structure of genes associated with MN by different chromosomes: (a) Chr2 (b) Chr3 (c) Chr9 (d) Chr11 (e) Chr19 (f) Chr22. Color scheme of linkage disequilibrium LD map is based on standard D’/LOD option in Haploview software, LD blocks calculated based on CI method.

Table 1 displays characteristics of genetic polymorphisms in MN research across three years in Taiwan. Genes were discussed previously, all involved in pathogenesis: *STAT4* , *TLR9* , *IL-6* , *TLR4* , *TRPC6* , *NPHS1* , and *MYH9* [8-14]. Our new information about polymorphisms on *MYD88, ACTN4,* and *KIRREL2* relates to MN susceptibility. Figure 1 shows distributions of genotypic and allelic frequencies of 27 polymorphisms on 10 genes in normal population in Taiwan. We observed rs3024908 polymorphism on STAT4 gene without G/G genotype; rs1060186 and rs12986337 on ACTN4 without A/A and C/C genotype, respectively; and rs2269530 on MYH9 without G/G genotype in normal population. We assessed genotypic and allelic frequencies of these in MN cases and controls (Table 2) to find strong links between MN and rs3024908 on *STAT4* gene, rs352139 on *TLR9* , rs1800796 on *IL-6* , rs10983755 and rs1927914 on *TLR4* , rs437168 on *NPHS1* , and rs443186 on *KIRREL2*. LD and Haplotype block structure were estimated via 27 polymorphisms on 10 MN-linked genes (Fig. 2). According to chromosome type, structures appeared as (a) Chr2 (b) Chr3 (c) Chr9 (d) Chr11 (e) Chr19 (f) Chr22. Color scheme of linkage disequilibrium (LD) map is based on standard D’/LOD option in Haploview software, LD blocks calculated by CI method.

**Table 1:** Characteristics of polymorphisms in study of idiopathic membranous nephropathy over these past three years in Taiwan.

Gene name	SNP database ID	Location	Variation Legend	Referneces
STAT4	rs3024908	2:191894141	3’UTR(G/T)	Chen et al., 2011 [8]
	rs3024912	2:191893087	3’UTR(A/G)	
	rs3024877	2:191904889	intro n 15(A/G)	
TLR9	rs352140	3:52256697	exon 2(C/T)	Chen et al., 2013 [9]
	rs352139	3:52258372	intro n 1 (C/T)	
MYD88^*^	rs7744	3:38184021	3’UTR(A/G)	
IL-6	rs1800796	7:22766246	C-572G	Chen et al., 2010 [10]
TLR4	rs10983755	9:120464670	5’UTR(A/G)	Chen et al., 2010 [11]
	rs1927914	9:120464725	5’UTR(A/G)	
	rs10759932	9:120465144	5’UTR(C/T)	
	rs11536889	9:120478131	3’UTR(C/G)	
TRPC6	rs3824935	11:101456002	3’UTR(C/T)	Chen et al., 2010 [12]
	rs17096918	11:101453995	intro n 1 (C/T)	
	rs4326755	11:101449358	intron 1 (A/G)	
NPHS1	rs401824	19:36342909	5’UTR(A/G)	Lo et al, 2010 [13]
	rs437168	19:36334419	exon 3(C/T)	
	rs3814995	19:36342212	exon 17(A/G)	
ACHT4^*^	rs1060186	19:39221295	3’TJTR(A/G)	
	rs3745859	19:39196745	exon 5(C/T)	
	rsl2986337	19:39215172	exon 16 (C/T)	
KIRREL2^*^	rs443186	19:36345951	5’UTR(A/C)	
	rs447707	19:36347400	5’UTR(A/G)	
	rs446014	19:36348078	exon 1(AJC)	
MYH9	rs12107	22:36677982	3’UTR(A/G)	Chen et al., 2013 [14]
	rs11703176	22:36678476	3’UTR(A/G)	
	rs2269530	22:36684358	3’UTR(A/C)	
	rs7078	22:36677914	exon 34(T/G)	

*: Unpublished new results by authors

## 3. Transducer and Activator of Transcription 4 (STAT4)

The signal transducer and activator of transcription 4 (*STAT4* ) gene, located on chromosome 2q32.2-32.3, encodes a transcription factor essential to inflammation in various immune-mediated diseases [15]. *STAT4* plays a key role in regulating immune response by transmitting signals activated in response to cytokines like Type 1 IFN, IL-12, and IL-23 [16]. *STAT4* is vital for IL-12 inducing naïve CD4+ T differentiation of into Th1 cells that drive chronic inflammation by secreting high levels of cytokines like IFN-γ and TNF-α [17]. *STAT4* haplotype characterized by rs7574865 exhibited strong linkage with rheumatoid arthritis (RA), systemic lupus erythematosus (SLE), and autoimmune disease: e.g., systemic sclerosis, Sjögren’s syndrome, Type 1 diabetes [18-22]. SY Chen et al. (2011) reported significant difference in genotype frequency at rs3024908 SNP in MN patients versus controls (p = 0.014); those with GG genotype at rs3024912 SNP face higher risk of kidney failure in MN cases (adjusted odds ratio [OR] = 3.255; 95% confidence interval [CI] = 1.155-9.176, p = 0.026) [8].

## 4. Toll-like receptor 9 (TLR-9)

Toll-like receptors (TLRs) play a central role in response of both the innate and adaptive immune system to microbial ligands [23]. Glomerular disease is triggered or exacerbated by microbes that activate the immune system by Toll-like receptor (TLR) ligation [24-25]. Exaggerated TLR activation associates with ischemic kidney damage, acute kidney injury, end-stage renal failure, acute renal transplant rejection, acute tubulointerstitial nephritis and delayed allograft function [25]. TLR9 is implicated in initiation and progression of kidney disease (human and experimental): e.g., crescetic, lupus nephritis, glomerulonephritis, IgA nephropathy [26-28]. YT Chen et al. (2013) cited AA genotype at rs352139 SNP or GG at rs352140 SNP indicating higher risk of MN (odds ratio [OR]=1.55; 95% confidence interval [CI]=1.02–2.35, at rs352139 SNP; OR=1.57; 95% CI=1.03–2.39, at rs352140 SNP); A-G haplotype raised susceptibility to decreased creatinine clearance rate and serious tubule-interstitial fibrosis [9].

## 5. Interleukin-6 (IL-6)

Mounting evidence hints pro-inflammatory cytokines like tumor necrosis factor and interleukin-1 (IL-I), playing a crucial role in lupus nephritis and proliferative IC glomerulonephritis [29-31]. Interleukin-6 (IL-6) is also implicated in manifestations of nephropathy [32-33]. Urinary IL-6 stimulated proliferation of rat mesangial cells yielding IL-6 *in vitro* [32]. Urinary IL-6 has been identified as a marker of renal IL-6 production [34]: high levels of it arise in 30-50% of IgA nephropathy cases [32-33]. IL-6 may thus act as an autocrine growth factor in the mesangium and dysregulated IL-6 production in mesangial proliferation linked with glomerulonephritis. Among Taiwan’s Han Chinese, data show starkly different genotype and allele frequency at IL-6 C-572G SNP in MN cases versus controls (p=1.6E-04 and 1.7E-04, respectively). People with C allele or with CC genotype at IL-6 C-572G SNP show higher risk of MN (OR=2.42 and 2.71; 95% CI=1.51-3.87 and 1.60-4.60, respectively) [10].

## 6. Toll-like receptors 4 (TLR-4)

Toll-like receptors (TLRs), a key element of human innate immune response, up-regulate proinflammatory cytokines and co-stimulatory molecules as a first line host defence [35]. TLRs are cited as key components of pathogen-recognition process mediating inflammatory response [36]. TLR4 interacts with ligands such as heat-shock proteins [37]; TLR4 polymorphisms reportedly link with inflammatory disease and/or cancer: e.g., Crohn’s disease, ulcerative colitis, cervical cancer [38-40]. Recent report indicated significant difference of TLR4 gene rs10983755 A/G (p < 0.001) and rs1927914 A/G (p < 0.05) polymorphisms between controls and MN patients. Distributions of rs10759932 C/T and rs11536889 C/T polymorphisms differed significantly. Higher triglyceride level arose in non-GG versus GG group. Genotype of non-AA had a far higher proteinuria ratio than AA group [11].

## 7. Nephrin (NPHS1)

This signaling adhesion protein is believed to play a vital role in modulating renal function [41]. Research on nephrin function initially focused on interaction of slit diaphragm structural components (SD) [42]. Recent research demonstrates nephrin as involved in signal processes critical to podocyte function, survival and differentiation [43]. Polymorphisms in NPHS1 demonstratrably play a pivotal role in progression of renal failure [44]. Mutations of NPHS1 or NPHS2 reportedly associate with severe nephrotic syndrome that progresses to end-stage renal failure in children [45]. R229Q, a NPHS1 variant, meant 20-40% higher risk of focal segmental glomerulosclerosis in European populations [46]. Lo et al., 2010 reported significant difference in genotype frequency distribution of rs437168 polymorphism between MN patients and controls. Their results also showed frequency of G allele significantly higher in the MN group; stratified analysis linked high disease progression in AA genotype of rs401824 and GG genotype of rs437168 patients with low rate of remission [13].

**Table 2:** Genotypic and allelic frequencies of polymorphisms in MGN patients versus controls.

		Genotype frequency							Allele frequency				
Gene	db SNP ID	Patient with MGN (%)			Control (%)			*p* value	Patient with MGN (%)		Central (%)		*p* value
		1	heter	2	1	heter	2		1	2	1	2	
STAT4 (Chr2)	rs3024903 (G/A)	4(2.9)	33(24.1)	100(73)	0(0)	77(29.1)	188(70.9)	**0.014** ^*^	41(15)	233(85)	77(14.5)	453(84.5)	0.869
	rs3024912 (T/G)	31(22,8)	7X55.1)	30(22.1)	66(25)	127(48.1)	71(26.9)	0,388	135(49.6)	137(50.4)	269(50.9)	259(49.1)	0.725
	rs3024877 (G/A)	36 (26.1)	70(50.7)	32(23.2)	61(23.1)	138(52J)	65(246)	0.797	142(51.4)	134(43.6)	260(49.2)	263(50.8)	0.552
TLR9 (Chr3)	rs352139 (G/A)	16 (11.9)	51 (38.1)	67(50.0)	28 (10 6)	133 (50 2)	104(393)	**0.067** ^*^	83(31.0)	185 (69D)	189 (35.7)	341 (64.3)	0.187
	rs35214Q (G/A)	70(523	48 (35.8)	16(11.9)	108(41.1)	127 (48,3)	28(10.6)	0J3J7	188 (70.1)	80(29.9)	343 (65.2)	183 (34.8)	0.162
MYD88 (Chr3)	rs7744(A/G)	44(331)	75(56.4)	14(10.5)	93(35.6)	137(52.5)	31(115)	0758	163(613)	103(38.7)	323(61.9)	199(38.1)	0.870
IL6 (Chr7)	rs1800796 (C/G)	84(79.2)	20 (18.9)	2(1-9)	155(585)	95 (35.8)	15(5.7)	**< 0.001** ^*^	188 (88.7)	24(11.3)	405 (76.4)	125(23.6)	**< 0.1101** ^*^
TLR.4(Chi9)	rs10983755 (A/G)	3(6.0)	75 (56.0)	51 (38.1)	12 (4 J)	98 (37.0)	155 (585)	**< 0.001** ^*^	91 (34.0)	177 (66.8)	122 (23.D)	408(77.0)	**0.001** ^*^
	rs1S27914(A/G)	44(32.8)	67 (50.0)	33(17.2)	121 (45 7)	104(392)	40(15.1)	**0.045** ^*^	155 (57.8)	113(42.2)	346 (65.3)	184(34.7)	**0.039** ^*^
	rs10759932 (C/T)	8(6.0)	56(41.8)	70(52.2)	12(4.6)	97(36 9)	154(58 6)	0.465	72(26.9)	196(73.1)	121(23.0)	405(77.0)	0.230
	rs11536889 (C/T)	6(4.5)	45 (33.8)	82(61.7)	11(4.2)	75 (28.7)	175 (67.0)	0.559	57(21.4)	209 (78 j6)	97(186)	425(81.4)	0.341
TRPC6 (Chrll)	rs3824935 (T/C)	0(0)	16(11.9)	118(88.1)	4(1.5)	43(16 J)	216(82.1)	0.126	252 (94.0)	16(6.0)	475(90.3)	52(97)	0.063
	rs17096918(C/T)	32(23.9)	74(55,2)	28(20.9)	64(24.2)	141(53.4)	59 (22.3)	0.930	138 (51.5)	130 (48.5)	269(50.9)	259(49.1)	0.834
	rs432S755(G/A)	25 (18.7)	74(55.2)	35(26.1)	39 (147)	134(50 6)	92 (34.7)	0.192	124(463)	144(53.7)	212(40.0)	318 (60.0)	0.090
NPHS1 (ChrlS)	rs401824(A/G)	113(81.9)	24(174)	1(07)	196(74.0)	63(23,8)	6(2.3)	0.158	250(90.6)	26(9.4)	455(35.8)	75(142)	0.054
	rs437168(A/G)	0(0)	25 (18.5)	110(81.5)	1 (0.4)	81 (30.6)	183 (69 1)	**0.026** ^*^	25(9.3)	245 (90.7)	83(15.7)	447 (84.3)	**0.012** ^*^
	rs3814995 (C/T)	18(136)	51(3Sj6)	63(47,7)	42(16.3)	106(41.2)	109(42 4)	0372	87(33.0)	177(67 0)	190(37.0)	324(63,0)	0.269
ACTN4(Chrl9)	rs10601S6 (G/A)	55(401)	32(599)	0	105(39.6)	160(60.4)	0	0919	192(70)	82(30)	370(69.9)	160(30.1)	0.939
	rs3745859 (T/C)	18(13.3)	73(53.7)	45(33)	44(16.7)	125(47.3)	95(36)	0.444	109(40)	163(60)	213(403)	315(59.7)	0.942
	rs12986337 (T/C)	125(92.6)	10(7.4)	0	242(91.7)	22(83)	0	0747	260(96.3)	10(3.7)	506(95.8)	22(4.2)	0.752
KIKREL2 (Chr 19)	rs443186 (C/A)	0(0)	26(19.1)	110(80.9)	5(1.9)	75 (28.5)	183 (69.6)	**0.015** ^*^	246(90.4)	26 (9.6)	441 (33.8)	85 (16.2)	**0.011** ^*^
	rs447707 (A/G)	83(619)	43 (32.1)	8(6.0)	138(52.1)	98 (37.0)	29(10.9)	0.103	209(78.0)	59 (22.0)	374(70.6)	156(294)	**0.026** ^*^
	rs446014(C/A)	0(0)	15(11.2)	119(88.8)	1 (0.4)	30(11.4)	233 (88.3)	0872	253 (94.4)	15(5.6)	496(94.0)	32 (6.0)	0.793
MYH9(Chr 22)	rs12107 (G/A)	9(6.7)	53(39.3)	73(54)	22(8.3)	132(49.8)	111(41,9)	0.069	71(26.3)	199(73.7)	176(33.2)	354(66.8)	**0.045** ^*^
	rs11703176 (C/A)	9(6.7)	0(0)	125(93.3)	22(83)	0(0)	243(91.7)	0J76	18(6.7)	250(93.3)	44(3.3)	486(91,7)	0.429
	rs2269530 (T/G)	51(38.1)	83(61.9)	0(0)	100(37.7)	165(62.3)	0(0)	0.950	185(69)	83(31)	36X68.7)	165(31.3)	0.963
	rs7078 (C/T)	115(85.8)	19(143)	0(0)	221(84.4)	42(15,6)	0(0)	0.640	249(92.9)	19(7.1)	434(924)	42(7.6)	0.654

## 8. Myosin Heavy Chain 9 (MYH9)

This gene, expressed in glomerular podocytes and mesangial cells, encodes nonmuscle myosin IIA [47, 48]. Currently, 44 of it mutations have been reported [49], possibly involving either N-terminal motor or C-terminal tail domain of MYH9 gene encoding for the heavy chain of nonmuscle myosin-IIA. The MYH9 haplotypes show replicated association with risk and protection [50, 51]. They are proven as associated with kidney disease in African Americans and European Americans [52, 53]; MYH9 also affects kidney function in Europeans [54]. Results portend statistically significant difference in allele frequency distribution at rs12107 between MN cases and controls (p = 0.04). Persons with AA genotype at rs12107 SNP who contract MN face higher risk of kidney failure than other MN cases (adjusted odds ratio: 1.63; 95% confidence interval: 1.08-2.48, p = 0.02). C-A haplotype is susceptible to MN [14].

## 9. Kin of IRRE Like 2 (Drosophila) (KIRREL2)

This protein exhibits sequence resembling that of several cell adhesion proteins: e.g., Drosophila RST (irregular chiasm C-roughest), mammalian KIRREL (akin to irregular chiasm C-roughest; NEPH1), NPHS1 (nephrin). The former, a complex gene with mutations originally assigned to separate loci, has alleles originally assigned to irregular chiasm locus, affecting axonal migration in the optic lobes. Other alleles, originally assigned to the roughest locus, link with reduced apoptosis in the retina, inducing roughened appearance of the compound eye [55] and [56]. The mammalian KIRREL/NEPH1 and NPHS1 genes both encode components of the glomerular slit diaphragm in kidneys [57], [58] and [59]. We noted significant difference in genotype frequency distribution of rs443186 polymorphism between MN patients and controls. Data showed frequency of C allele at rs443186 and A allele at rs447707 definitely higher in the MN group. Data indicate individuals with AA genotype at rs443186 SNP face higher risk of MN (Table 2).

## 10. Conclusion

Genetic susceptibility plays a major role in pathogenesis [60]. Research efforts, including GWASs, have been invested worldwide to identify susceptibility genes for several diseases. GWASs are considered as a powerful and promising approach [61]. Candidate gene approach along with an appropriate analysis remains the method of choice to evaluate genes of interest conferring susceptibility to specific disease. Most genes contributing to MN susceptibility remain unidentified; well-organized approach like GWASs may obtain definite conclusions regarding such genes in the near future.

## Acknowledgements

This study is supported in part by Taiwan Ministry of Health and Welfare Clinical Trial and Research Center of Excellence (DOH102-TD-B-111-004), China Medical University Hospital (DMR-102-086) in Taiwan.
